# Comparison of Labor and Delivery Complications and Delivery Methods Between Physicians and White-Collar Workers

**DOI:** 10.3390/ijerph17145212

**Published:** 2020-07-19

**Authors:** Chun-Che Huang, Wen-Feng Lee, Ching-Hsueh Yeh, Chiang-Hsing Yang, Yu-Tung Huang

**Affiliations:** 1 Department of Healthcare Administration, I-Shou University, Kaohsiung 82445, Taiwan; ajerhuang@isu.edu.tw; 2 Department of Obstetrics and Gynecology, Chang Gung Memorial Hospital, Linkou Medical Center, Taoyuan 33302, Taiwan; wanna0507@gmail.com; 3 School of Nursing, Kaohsiung Medical University, Kaohsiung 80708, Taiwan; chyeh@kmu.edu.tw; 4 Department of Health Care Management, College of Health Technology, National Taipei University of Nursing Health Sciences, Taipei 10845, Taiwan; yangch@ntunhs.edu.tw; 5 Center for Big Data Analytics and Statistics, Chang Gung Memorial Hospital, Linkou Medical Center, Taoyuan 33302, Taiwan

**Keywords:** physicians, white-collar workers, labor complications, delivery

## Abstract

To evaluate labor and delivery complications and delivery modes between physicians and white-collar workers in Taiwan, this retrospective population-based study used data from Taiwan’s National Health Insurance Research Database. We compared 1530 physicians aged 25 to 50 years old who worked and had singleton births between 2007 and 2013 with 3060 white-collar workers matched by age groups, groups of monthly insured payroll-related premiums, previous cesarean delivery, perinatal history anemia, and gestational diabetes mellitus. The logistic regression models were used to assess the labor and delivery complications between the two groups. Multivariate analysis revealed that physicians had a significantly higher risk of placenta previa (odds ratio (OR) 1.35, 95% confidence interval (CI) 1.08–1.69) and other malpresentation (OR 1.86, 95% CI 1.45–2.39) than white-collar workers, whereas they had a significantly lower risk of placental abruption (OR 0.53, 95% CI 0.40–0.71), preterm delivery (OR 0.75, 95% CI 0.61–0.92), and premature rupture of membranes (OR 0.72, 95% CI 0.59–0.88). Increased risks of some adverse labor and delivery complications were observed among physicians, when compared to white-collar workers. These findings suggest that working women should take preventative action to manage occupational risks during pregnancy.

## 1. Introduction

Physicians often work in environments where they are exposed to various occupational and environmental hazards, including radiation, cytotoxic drugs, anesthetic gases, and infectious materials or blood [1,2,3]. In addition, physicians also entail long working hours, extended work shifts, and a high workload, thereby they encounter more psychologically and physically stressful conditions [4,5], especially the increasing proportion of female doctors [6]. The impact of these exposures may increase the risks of maternal and fetal complications during pregnancy and delivery [1,2,4,5,6,7]. In addition, female physicians’ medical knowledge and practice in a profession may influence their health-related behaviors and their use of healthcare services [6,7]. It is therefore important to assess how pregnant physicians have adverse outcomes of pregnancy and actual delivery modes.

To the best our knowledge, prior research has shown evidence of the risk of adverse pregnancy outcomes among resident physicians or specialists in different settings, including medical schools [4], anesthetic [6], orthopedic [8], urology [9], and obstetrics and gynecology departments [10]. However, the results are not controversial. A few survey research studies reported that among anesthetic trainees [6], orthopedic surgeons [8], urologists [9], and residents [4,10] had a higher rate of pregnancy and obstetric complications as compared with anesthesiologists, general women, and spouses or partners of male residents, respectively. These studies were based on regional samples, limited to selected hospitals, and less relevant to adjustment. However, there is little or no significant differences in the prevalence of miscarriage, ectopic gestations, stillbirths, placenta previa, preeclampsia, and prematurity between physicians [7,11] or residents [12] and other women of a similar socioeconomic background. We therefore conducted a nationwide population-based study in Taiwan to evaluate labor and delivery complications and delivery modes among physicians who worked in a profession in comparison with white-collar workers who are not medical personnel.

## 2. Materials and Methods

### 2.1. Data Sources and Study Population

Data in this population-based cross-sectional study were retrieved from the National Health Insurance Research Datasets (NHIRD) in Taiwan covering the period from 2007 to 2013. The obtained data included inpatient expenditures by admissions, registry for beneficiaries, registry for medical personnel, and registry for contracted medical facilities. All encrypted datasets for the relevant variables were interlinked via the identification numbers for woman, medical personnel or medical institution. Protocol for the confidentiality of data and privacy protection was strictly followed before data was released to researchers. The procedural and diagnostic codes for each instance of hospitalization were categorized according to the diagnosis-related groups (DRG) or International Classification of Diseases, Ninth Revision, Clinical Modification (ICD-9-CM) coding system. The National Health Insurance Administration (NHIA) mandated the requirements for standardized procedures and performed expert reviews to ensure quality of care and the accuracy of claim files. This study was approved by the Institutional Review Board of the Kaohsiung Medical University Hospital, Taiwan (KMUH-IRB-20150017). Written consent from study objects was not obtained because the NHIRD consists of de-identified secondary data for research purposes.

On the basis of the NHIA’s DRG case payment system, delivery mode was identified by the DRG and procedure codes and were classified as follows: vaginal delivery (DRG 0373A or 0373C) and cesarean delivery (0371A or 0373B). We identified 1664 physicians and 465,380 white-collar workers with a singleton birth who aged 25‒50 years and had either vaginal or cesarean delivery between 2007 and 2013. Very few individuals qualify as practicing physicians prior to the age of 25 years, and pregnant women aged over 50 years are atypical. Six (<0.01%) births were excluded due to incomplete information regarding attending obstetrician gender and age. In addition, we also excluded 134 (8.1%) physicians who did not work in a profession during the study period. We used frequency matching to minimize the differences in age groups, groups of monthly insured payroll-related premiums, previous cesarean delivery, perinatal history anemia, and gestational diabetes mellitus (GDM) between the two groups. White-collar workers (defined as the control group) were sorted in random order and reviewed in sequence until the required number of controls met matching criteria for inclusion at a ratio of 1:2. Our final sample included 1530 physicians and 3060 white-collar workers with a singleton birth who were not medical personnel.

### 2.2. Measures

The main outcomes measured were the appearances of labor and delivery complications and delivery method between physicians and white-collar workers based on previous research and information available in the NHIRD. The labor and delivery conditions were selected on the basis of previous studies [13,14] and were as follows: placenta previa (ICD-9-CM codes: 641.0, 641.1 and 762.0), placental abruption (641.2 or 762.1), eclampsia and severe preeclampsia (642.4, 642.5 and 642.7), breech presentation (652.1, 652.2, 660.0, 761.7 and 763.0), other malpresentation (652.3, 652.4, 652.6‒652.9 or 763.1), preterm delivery (<37 weeks of gestation, 644.0‒644.2), premature rupture of the membrane (PROM; 658.1 or 658.2), post-term delivery (≥42 weeks of gestation; 645), and antepartum or postpartum hemorrhage (641.3, 641.8, 641.9, and 666.0‒666.3). In addition, delivery methods were divided into vaginal (DRG codes 0373A or 0373C) versus cesarean delivery (0371A or 0373B) based on the NHIA’s DRG codes.

Occupational information was obtained from the registry for medical personnel and the registry for beneficiary files. Physicians who worked in a profession were identified as a study group, while other white-collar workers were identified as a comparison group. Only women who had full-time employment were included, and they were categorized as physicians (doctors of western medicine only, traditional Chinese doctors, and dentists were excluded) and white-collar workers who were employed full-time and worked for government agencies or private institutions. The latter group was selected by using frequency matching on the demographics and clinical characteristics as a comparison group.

Covariates were selected according to prior research and information available in the NHIRD database and were as follows: characteristics of mothers (including age, monthly insured payroll-related premiums, previous cesarean delivery, and major pregnancy complications), hospitals (including accreditation level, ownership, and geographic region), attending obstetricians (including gender and age), and year of labor.

Insured payroll-related premium was obtained from the registry for beneficiaries files. The insurable income was classified into three groups: > New Taiwanese dollars (NT$) 72,800, 36,301–72,800, and ≤36,300 per month. Additionally, previous cesarean delivery (ICD-9-CM code 654.2), perinatal history anemia (280, 281, 285.9 or 648.2), and gestational diabetes mellitus (GDM; 648.0, 648.8 or 775.0) [14] were selected as covariates. Maternal anemia and GDM are the most common pregnancy-related conditions, which are associated with an increased risk of labor and delivery complications [15].

The registry for contracted medical facilities provided information related to accreditation level, ownership, and the geographic location of the medical institutions in which deliveries were performed. According to the hospital accreditation system in Taiwan, medical centers were the hospitals with better medical care and teaching ability, followed by regional hospitals, local hospitals, and obstetrics/gynecology clinics. Hospital ownership was categorized into public, private for-profit, and non-profit proprietary hospitals. Location of service was categorized into four regions (northern, central, southern, and eastern) according to the National Statistics of Regional Standard Classification. The registry for medical personnel provided information on the gender and age of attending obstetricians.

### 2.3. Statistical Analysis

All analyses were conducted by using SAS version 9.4 (SAS Institute, Cary, NC, USA). We adopted frequency matching to balance the observed covariates between physicians and white-collar workers and to minimize the selection bias. The demographic characteristics, occurrence of labor and delivery complications, and the delivery methods between the two groups were examined by using the Kruskal–Wallis test for continuous variables and the chi-square or Fisher’s exact tests for categorical variables, whichever was appropriate. Univariate and multivariate logistic regression analyses were used to estimate the difference in labor and delivery complications and delivery mode between the two groups with singleton births. The analysis was performed to calculate crude and adjusted odds ratios (OR) and 95% confidence intervals (CIs) of the presence of labor and delivery complications and delivery methods among pregnant physicians who worked in a profession. A *p* < 0.05 was considered statistically significant.

## 3. Results

Table 1 shows the basic description of the study population characteristics. A comparison of characteristics between practicing physicians and white-collar workers showed significant differences in age, monthly insured payroll-related premiums, perinatal history anemia, and year of labor before frequency matching (Table S1). After matching at a ratio of 1:2, the two groups were well balanced for maternal age, monthly insured payroll-related premiums, previous cesarean delivery, perinatal history anemia, and GDM (all *p* = 1.000). In terms of hospital and obstetrician characteristics, the pregnant physicians were more likely than pregnant white-collar workers to be admitted to medical centers (*p* < 0.001). They also delivered more frequently in public or non-profit proprietary institutions (*p* < 0.001). The locations of the facilities where the women gave birth significantly differed between the two groups. In addition, the pregnant physicians were more commonly delivered by female (*p* < 0.001) and younger attending obstetricians (*p* < 0.001). The distribution of the year-of-labor between the two groups declined from the year 2007 to 2010, and then increased to 2012, before declining.

The occurrence of placenta previa (2.9% vs. 2%), other malpresentation (1.2% vs. 0.8%), post-term delivery (12.6% vs. 11.4%), and antepartum or postpartum hemorrhage (3.1% vs. 2.6%) were not statistically significantly higher among physicians compared with white-collar workers (Table 2). However, after matching, physicians had significantly lower rate of cesarean delivery than white-collar workers (31.1% vs. 35.1%; *p* = 0.008).

After adjustment for maternal demographics and institutional and obstetrician characteristics, multivariate results indicated that physicians were found to be at a significantly higher risk of placenta previa (OR 1.35, 95% CI 1.08–1.69) and other malpresentation (OR 1.86, 95% CI 1.45–2.39) compared with white-collar workers (Table 3). However, they had a significantly lower risk of placental abruption (OR 0.53, 95% CI 0.40–0.71), preterm delivery (OR 0.75, 95% CI 0.61–0.92), and PROM (OR 0.72, 95% CI 0.59–0.88) than white-collar workers after adjustments. Nevertheless, after multivariate adjustments, the difference in cesarean delivery became not statistically significant between the two groups.

## 4. Discussion

Our study indicated that after matching and adjusting for relevant covariates, physicians exhibited a significantly increased risk of placenta previa and other malpresentations during labor and delivery as compared with white-collar workers, whereas they had a lower risk of placental abruption, preterm delivery, and PROM.

Prior experiences of complicated birth or previous cesarean delivery may increase the risks of late pregnancy complications of placenta previa and other malpresentations among physicians. In addition, in this present study, a greater rate of antepartum or postpartum hemorrhage was also observed among physicians, but the difference was not statistically significant. Our finding is inconsistent with previous studies [12] that indicated that there was no significant difference in the rates of placenta previa, preterm labor, and PROM between residents and general women. However, prior studies reported women with comorbid conditions of diabetes and hypertension had increased risks of placenta previa, placental abruption, and malpresentations [16,17]. Furthermore, a higher prevalence of abnormal presentations could be due to the occurrence of pendulous abdomen, or increased occurrence of pelvic inclination or placenta previa [17]. The majority of other malpresentations were delivered by cesarean delivery [18]. Therefore, pregnant physicians with prior complicated pregnancies may have increased risks of placenta previa and other malpresentations.

Lower occurrence of placental abruption, preterm delivery, and PROM among physicians might be associated with their medical knowledge and awareness of obstetrical conditions. Johnson and Rehavi (2016) presented that physician-mothers could differ in their risk preferences or in their professional knowledge and ability to evaluate treatment options and implications during pregnancy and labor [19]. However, the observed differences between the two groups may not alone be responsible for professional knowledge and ability. In addition, we cannot totally rule out the possibility that obstetricians may treat physician-mothers differently out of professional courtesy [19] and physician-mothers may have better access to quality care [20]. Thus, physician’s medical knowledge and access to quality care might partially explain their lower occurrence of placental abruption, preterm delivery, and PROM.

Although actual reasons for the differences in the risks of labor and delivery complications between physicians and white-collar workers have yet to be verified, it may be partially attributed to discrepancy in occupational and environmental exposures. An increased risk of pregnancy hypertension, placental abruption, and low birth weight among physicians has been reportedly associated with a heavy workload and a higher psychological stress than that which occurs during resident [10,12] or attending physician pregnancies [1,3,4]. This reveals that the particular labor and delivery complications of physicians may be aggravated by potential occupational and environmental exposures [7]. These cumulative exposures may influence women’s reproductive health, which may increase risks of spontaneous abortion, reduced intrauterine growth, preterm delivery, and low birth weight [8,11,12]. Several obstetric conditions such as prior cesarean delivery, maternal anemia, gestational diabetes, abnormal presentations, placenta previa, placental abruption, and prematurity were related to an increased use of cesarean deliveries [21]. Thus, we considered the possibility that physicians might have been exposed to occupational and environmental hazards during labor and delivery, which may have contributed to the results.

Our result indicated that a lower rate of undergoing cesarean delivery was observed among physicians, which is consistent with prior research [19,22]. The plausible reason for this may be that physicians in specialties with sufficient medical training and knowledge have the potential to know which treatment is appropriate for them during labor and delivery [19,20]. In addition, there is less of an information asymmetry about childbirth between physician-mothers and obstetricians and this makes them less susceptible to potential risk taking at delivery [19]. Therefore, the lower rate of cesarean delivery among physicians may due to their relevant expertise in medicine and access to better information about potential delivery complications. Otherwise, we also found that physicians were more likely than white-collar workers to choose medical centers as a birth place. They were also more likely to deliver in public or in non-profit proprietary institutions. The women’s preferences for birth settings might explain the differences in use of cesarean delivery between the two groups.

This is the first nationwide population-based study to delineate labor and delivery complications among physicians who worked in a profession in Taiwan, as compared with white-collar workers who are not medical personnel. This study makes a valuable contribution to the assessment of labor complications and actual delivery mode among physicians who worked in a profession, rather than simply reports on the attitudes and self-report measures of risks of obstetric events and various modes of delivery, which are not necessarily an accurate reflection of labor and delivery conditions. Despite the limited interpretation of the findings, it is important to understand the differences in labor and delivery complications between practicing physicians and other white-collar workers who are not medical personnel. However, the multifactorial causes of adverse labor and delivery conditions are complex, and the present study highlighted several areas where future research may illuminate some of the findings.

Several limitations of this study should be noted. First, owing to data limitation, results may be confounded by other unmeasured factors, including labor induction, pre-gestational body mass index (BMI), and obstetric pathology (pre-eclampsia) that may be associated with the end of cesarean delivery. Second, white-collar workers may differ from physicians regarding unmeasured socioeconomic variables such as education and working conditions, which may influence the observed associations. Therefore, as the comparison group, female white-collar workers were selected by using frequency matching with age groups, groups of monthly insured payroll-related premiums, previous cesarean delivery, perinatal history anemia, and GDM. Third, detailed information on chemical, physical, biological, and psychosocial exposures at work and other lifestyle variables were not available, all of which could be related to the risk of labor and delivery complications. Thus, a more-sophisticated analysis is needed to better understand this association under consideration.

## 5. Conclusions

In summary, the present study reveals that pregnant physicians who worked in a profession are at a higher risk of placenta previa and other malpresentations as compared with pregnant white-collar workers, whereas they are at a lower risk of placental abruption, preterm delivery, and PROM. The magnitude of occupational exposures and working conditions for pregnant physicians may potentially contribute to their risk of these labor and delivery complications. However, there are likely other unobserved factors on which pregnant physicians differ. The finding illustrates that working women should take preventative action to manage occupational risks, and that this is especially necessary in late pregnancy and delivery.

## Figures and Tables

**Table 1:** Distribution of maternal demographics and institutional and obstetrician characteristics between practicing physicians and white-collar workers with singleton births, 2007‒2013 (*n* = 4590).

Characteristics	Physicians		White-Collar Workers		*p*
	(*n* = 1530)		(*n* = 3060)		
Maternal age (years), mean (SD)	32.7	(3.3)	32.9	(3.4)	1.000 ^†^
25–29.9	236	(15.4)	472	(15.4)	1.000 ^‡^
30–34.9	903	(59.0)	1806	(59.0)	
≥35	391	(25.6)	782	(25.6)	
Monthly insured payroll-related premiums (NT$)					1.000 ^‡^
>72,800	855	(55.9)	1710	(55.9)	
36,301–72,800	591	(38.6)	1182	(38.6)	
≤36,300	84	(5.5)	168	(5.5)	
Previous cesarean delivery	122	(8.0)	244	(8.0)	1.000 ^§^
Perinatal history anemia	78	(5.1)	156	(5.1)	1.000 ^§^
Gestational diabetes mellitus	15	(1.0)	30	(1.0)	1.000 ^§^
Institutional accreditation level					<0.001 ^‡^
Medical center	1111	(72.6)	865	(28.3)	
Regional hospital	230	(15.0)	877	(28.7)	
Local hospital	91	(6.0)	729	(23.8)	
Obstetrics/gynecology clinic	98	(6.4)	589	(19.2)	
Institutional ownership					<0.001 ^‡^
Public	512	(33.5)	383	(12.5)	
Private for-profit	208	(13.6)	1323	(43.2)	
Non-profit proprietary	810	(52.9)	1354	(44.3)	
Geographic location of institutions					<0.001 ^‡^
Northern	831	(54.3)	2266	(74.0)	
Central	262	(17.1)	338	(11.1)	
Southern	421	(27.5)	441	(14.4)	
Eastern	16	(1.1)	15	(0.5)	
Obstetrician gender					<0.001 ^§^
Male	1262	(82.5)	2701	(88.3)	
Female	268	(17.5)	359	(11.7)	
Obstetrician age (years), mean (SD)	45.7	(6.7)	47.3	(6.9)	<0.001 ^†^
Year of labor					0.620 ^‡^
2007	252	(16.5)	518	(16.9)	
2008	249	(16.3)	468	(15.3)	
2009	220	(14.4)	403	(13.2)	
2010	175	(11.4)	326	(10.7)	
2011	194	(12.7)	427	(13.9)	
2012	232	(15.1)	471	(15.4)	
2013	208	(13.6)	447	(14.6)	

Note: ^†^ Kruskal–Wallis test; ^‡^ chi-square test; ^§^ Fisher’s exact test; NT$, New Taiwan dollars; SD, standard deviation.

**Table 2:** Labor and delivery complications and delivery methods between practicing physicians and white-collar workers with singleton births, 2007‒2013 (*n* = 4590).

Characteristics	Physicians		White-Collar Workers		*p* ^§^
	(*n* = 1530)		(*n* = 3060)		
Placenta previa	44	(2.9)	61	(2.0)	0.074
Placental abruption	7	(0.5)	24	(0.8)	0.253
Eclampsia and severe preeclampsia	21	(1.4)	42	(1.4)	1.000
Breech presentation	126	(8.2)	256	(8.4)	0.910
Other malpresentation	18	(1.2)	26	(0.8)	0.335
Preterm delivery	77	(5.0)	154	(5.0)	1.000
PROM	106	(6.9)	251	(8.2)	0.144
Post-term delivery	193	(12.6)	348	(11.4)	0.225
Antepartum or postpartum hemorrhage	48	(3.1)	79	(2.6)	0.294
Cesarean delivery	476	(31.1)	1073	(35.1)	0.008

Note: ^§^ Fisher’s exact test; PROM, premature rupture of membranes.

**Table 3:** Univariate and multivariate analyses for labor and delivery complications and delivery methods among pregnant physicians (*n* = 4590).

Characteristics	Crude OR	(95% CI)	*p*	Adjusted OR ^†^	(95% CI)	*p*
Placenta previa	1.46	(0.98–2.16)	0.061	1.35	(1.08–1.69)	0.009
Placental abruption	0.58	(0.25–1.35)	0.208	0.53	(0.40–0.71)	<0.001
Eclampsia and severe preeclampsia	1.00	(0.59–1.70)	1.000	0.80	(0.63–1.01)	0.061
Breech presentation	0.98	(0.79–1.23)	0.880	1.14	(0.93–1.40)	0.207
Other malpresentation	1.39	(0.76–2.54)	0.286	1.86	(1.45–2.39)	<0.001
Preterm delivery	1.00	(0.76–1.32)	1.000	0.75	(0.61–0.92)	0.006
PROM	0.83	(0.66–1.06)	0.129	0.72	(0.59–0.88)	0.001
Post-term delivery	1.13	(0.93–1.36)	0.219	0.99	(0.82–1.18)	0.872
Antepartum or postpartum hemorrhage	1.22	(0.85–1.76)	0.280	1.04	(0.83–1.30)	0.761
Cesarean delivery	0.84	(0.73–0.95)	0.008	0.96	(0.81–1.15)	0.665

Note: ^†^ The model adjusted for maternal age, monthly insured payroll-related premiums, previous cesarean delivery, perinatal history anemia, gestational diabetes mellitus (GDM), institution- and obstetrician-related factors, and year of labor as compared with pregnant white-collar workers; CI, confidence interval; OR, odds ratio; PROM, premature rupture of membranes.

## Supplementary Materials

The following are available online at https://www.mdpi.com/1660-4601/17/14/5212/s1, Table S1. Characteristics of maternal demographics between practicing physicians and white-collar workers before and after frequency matching.
