# Supplementary material for: Comparison of Labor and Delivery Complications and Delivery Methods Between Physicians and White-Collar Workers
Source: Int J Environ Res Public Health. 2020 Jul 19;17(14):5212. doi: 10.3390/ijerph17145212 (PMC7400700; doi:10.3390/ijerph17145212)
Supplement: Supplementary file 1 [file ijerph-17-05212-s001.pdf]

International Journal of
Environmental Research and Public Health

MDPI

Supplementary Materials

Table S1. Characteristics of maternal demographics between practicing physicians and white-collar workers with singleton births before and after frequency matching, 2007–2013.

Characteristics	Before Matching					After Matching				
	Physicians (*n* = 1530)		White-Collar Workers (*n* = 415,341)		*p*	Physicians (*n* = 1530)		White-Collar Workers (*n* = 13,986)		*p*
Maternal age (years), mean (SD)	32.7	(3.3)	31.7	(3.7)	<0.001 †	32.7	(3.3)	32.9	(3.4)	1.000 †
25–29.9	236	(15.4)	126,913	(30.5)	<0.001 ‡	236	(15.4)	472	(15.4)	1.000 ‡
30–34.9	903	(59.0)	199,209	(48.0)		903	(59.0)	1806	(59.0)	
≥35	391	(25.6)	89,219	(21.5)		391	(25.6)	782	(25.6)	
Monthly insured payroll-related premiums (NT$)										1.000 ‡
>72,800	855	(55.9)	10,039	(2.4)	<0.001 ‡	855	(55.9)	1710	(55.9)	
36,301–72,800	591	(38.6)	140,903	(33.9)		591	(38.6)	1182	(38.6)	
≤36,300	84	(5.5)	264,399	(63.7)		84	(5.5)	168	(5.5)	
Previous cesarean delivery	122	(8.0)	33,346	(8.0)	1.000 §	122	(8.0)	244	(8.0)	1.000 §
Perinatal history anemia	78	(5.1)	16,310	(3.9)	0.025 §	78	(5.1)	156	(5.1)	1.000 §
Gestational diabetes mellitus	15	(1.0)	5858	(1.4)	0.190 §	15	(1.0)	30	(1.0)	1.000 §
Year of labor					0.004 ‡					0.620 ‡
2007	252	(16.5)	63,868	(15.4)		252	(16.5)	518	(16.9)	
2008	249	(16.3)	59,977	(14.4)		249	(16.3)	468	(15.3)	
2009	220	(14.4)	54,563	(13.2)		220	(14.4)	403	(13.2)	
2010	175	(11.4)	42,869	(10.3)		175	(11.4)	326	(10.7)	
2011	194	(12.7)	61,974	(14.9)		194	(12.7)	427	(13.9)	
2012	232	(15.1)	73,160	(17.6)		232	(15.1)	471	(15.4)	
2013	208	(13.6)	58,930	(14.2)		208	(13.6)	447	(14.6)	

Note: † Kruskal–Wallis test; ‡ chi-square test; § Fisher's exact test; NT$, New Taiwan dollars; SD, standard deviation.
